# How Residing in a Long-Term Care Facility Affects Suicidal Risk in Patients With Dementia: A Systematic Review

**DOI:** 10.7759/cureus.27858

**Published:** 2022-08-10

**Authors:** Narges Joshaghani, Nicole Villa, Omar Badla, Raman Goit, Samia E Saddik, Sarah N Dawood, Ahmad M Rabih, Ahmad Mohammed, Aishwarya Raman, Manish Uprety, Maria Jose Calero, Maria Resah B Villanueva, Safeera Khan

**Affiliations:** 1 Psychiatry and Behavioral Sciences, California Institute of Behavioral Neurosciences & Psychology, Fairfield, USA; 2 Internal Medicine, California Institute of Behavioral Neurosciences & Psychology, Fairfield, USA; 3 General Surgery, California Institute of Behavioral Neurosciences & Psychology, Fairfield, USA; 4 Internal medicine, California Institute of Behavioral Neurosciences & Psychology, Fairfield, USA; 5 Pediatrics, California Institute of Behavioral Neurosciences & Psychology, Fairfield, USA; 6 Internal Medicine, California Institute of Behavioral Neurosciences & Psychology, Fairfields, USA; 7 Obstetrics and Gynecology, California Institute of Behavioral Neurosciences & Psychology, Fairfield, USA; 8 Research, California Institute of Behavioral Neurosciences & Psychology, Fairfield, USA

**Keywords:** self-injurious behavior, long-term care facility, nursing home, suicide, dementia

## Abstract

This study aims to review the current literature regarding the association between suicide risk in patients aged 65 years or over with dementia residing in long-term care facilities (LTCs). We also evaluate the most common methods of suicide and protective versus risk factors of nursing home (NH) life on suicide behavior in patients with dementia.

Following preferred reporting items for systematic reviews and Meta-Analyses (PRISMA) guidelines, we performed a systematic review of the relevant free full-text articles found in PubMed, Pub Psych, Cochrane library, and Science Direct up until April 4, 2022. Medical Subject Heading (MeSH) terms and keywords (nursing home, long-term care facility, suicide, self-injurious behavior, dementia), were used to search for full-text randomized clinical trials (RCTs), cross-sectional, case-control, cohort studies, systematic reviews, and studies published in the English language in the last 12 years, focused on human subjects 65 years and older were selected based on predefined eligibility criteria. The search yielded 57,909 articles, of which 12 studies met our inclusion criteria. The articles were subjected to quality appraisal by two reviewers. We used the Newcastle Ottawa scale (NOS) for quality assessment with a mean score of six for 12 observational studies used in this paper.

Of the included reports, six were cross-sectional, five were cohort, and one was case-control. Four articles carefully examine the relationship between dementia and suicide, and all confirm the hypothesis that staying in LTCs reduces the risk of suicide in patients with dementia. However, the rest of the articles generally determine a higher risk of suicide in demented patients and describe male gender, non-Hispanic white race, younger age, newly diagnosed with dementia within one-year, mild dementia, comorbidities, depression, previous history of suicidal behavior, low social support and unstable family relationship as the risk factors of suicide in this population. In comparison, extended stay in NHs and other kinds of LTCs, severe dementia with impaired insight, older age, comorbid schizophrenia, physical disability with limitation and more difficulty preparing and executing a suicide plan, positive and robust social relationships, access to professional caregivers and high frequency of visits from relatives marked as the protective factors.

Existing research on suicide risk in long-term care facility residents with dementia is limited. However, due to the increase in dementia rates that require people to reside in NHs and on the other hand, considering the multiple risk factors of suicide in the elderly living in such places, the need for a screening system for identifying people at suicide risk and performing preventive therapeutic and behavioral interventions is well felt.

## Introduction and background

Dementia (also known as major neurocognitive disorder) is characterized by memory loss (initially of recent events), loss of executive function (such as the ability to make decisions or sequence complex tasks), other cognitive deficits, and changes in personality. This decline must be serious enough to affect social or occupational functioning, and reasonable attempts must be made to exclude other common conditions, such as depression and delirium. The most common types of dementia are Alzheimer's disease, vascular dementia, Lewy body dementia, and frontotemporal dementia. In all types of dementia, people will experience problems and cognitive functioning and are likely to experience behavioral and psychological symptoms of dementia [1]. The number of patients with dementia worldwide is expected to increase from about 50 million today to 152 million in 2050 [2].

To date, several risk factors for later-life suicide have been identified, the most significant of which is the presence of a major psychiatric illness and major depression most strongly associated with suicide in the elderly, although psychotic disorders, anxiety disorders, and substance abuse also increased risk, as does poor physical health status and impaired functional capacity. Dementia is a prevalent disorder of later life and is associated with neuropsychiatric symptoms such as depression, psychosis, and anxiety that have been identified as suicide risk factors [3]. On the other hand, cognitive deficits in dementia likely increase vulnerability to suicide by impairing sound decision-making. Moreover, they likely play a significant role in geriatric suicide due to average age-related cognitive decline and dementia neuropathology [4,5].

Since severe dementia could protect against suicide death by decreasing a person’s capacity to implement a suicide plan, patients with early dementia may have better cognition, giving them more sustained insight into their disease and better enabling them to carry out a suicide plan [6,7]. Also, increased impairment in cognition and personal activities of daily living is associated with a greater risk of NH admission [8]. Nursing homes have become increasingly important in end-of-life (EOL) care settings in the United States, especially for people with dementia. While 28% of non-traumatic deaths among people aged 65 years and older occur in a nursing home, about 67% of those aged 65 years and older, with a diagnosis of dementia, die in a nursing home. Hence, nursing homes are the final care settings for 70% of Americans dying from dementia [9].

There are more than 15,000 nursing homes and 28,000 assisted living communities nationwide. According to Alzheimer’s disease facts and figures report, 48 percent of nursing home residents live with Alzheimer’s or other dementias. In addition, among older adults in residential facilities, including assisted living, 42% or more have some form of Alzheimer’s or other dementias [10].

Risk factors for suicide, such as social isolation, depression, and functional impairment, are common among long-term care (LTC) residents. Therefore, these facilities may be necessary for preventing suicide among older adults [11-13].

Self-injurious behavior (SIB) in older adults is defined as harm inflicted on oneself without conscious suicidal intent. SIB as a separate entity distinct from suicidal intent is poorly understood. However, it is of great concern to the patient's families and caregivers, posing serious clinical challenges for clinicians. Self-injurious behavior (SIB) is a prevalent phenomenon reported as high as 14% in one study of nursing home subjects aged 65 and older. It is reported to be strongly associated with dementia and a risk of accidental death. It has been suggested that SIB among demented patients occurs in the context of poor impulse control and physical isolation which is mostly seen in the nursing home setting [14].

Since there are a considerable number of patients with dementia among nursing home residents and on the other hand, living in such places is itself a risk factor for suicide, this study aims to answer the effects of living in a nursing home on suicidal risk in patients with dementia. We also collected promotive or protective factors of suicidal risk in patients with dementia, which has been considered in recent 12-year studies in order to apply in future studies. This study is the first paper to systematically review the risk of suicide in patients with dementia residing in nursing homes along with its protective and exacerbating factors. 

## Review

Methods

Protocol

In order to achieve a high standard of reporting, we used the preferred reporting items for systematic reviews and meta-analyses (PRISMA) 2020 guidelines [15]. Before the search of the databases, a protocol was made and shared with the research team to analyze and finalize. The main question of the review: what is the effect of nursing home residing on suicidal risk in patients with dementia? what are protective and promotive factors of nursing home life on suicidal risk in patients with dementia? The impact of patient, intervention, comparison, outcome (PICO) strategy was used to formulate the question of this review. The review protocol can be acquired with a request addressed to the lead author.

Eligibility Criteria

The inclusion and exclusion criteria for screening studies are shown in Table 1.

**Table 1:** Inclusion and exclusion criteria

Inclusion criteria	Exclusion criteria
Ages ≥ 65-year old	Ages < 65-year old
Papers from April 2010 onward	Papers published before April 2010
Papers published in the English language	Papers not published in English
Papers focusing on human subjects	Studies that used animals
Randomized clinical trials (RCTs), case-control, cross-sectional, cohort studies, systematic reviews	Unpublished literature, books, and documents, grey literature

Information Sources

We searched four major electronic databases comprising medical and social science research (PubMed, Pub Psych, Cochrane library, and Science Direct) for titles and abstracts for April 4, 2010 - April 4, 2022.

The results of the search, keywords, and Medical Subject Heading (MeSH) terms used are shown in Table 2.

**Table 2:** Keywords, MeSH terms used, and results Medical Subject Headings (MeSH)

Keywords	Database	Initial Results	After screening	Eligible
Dementia [MeSH] AND Suicide [MeSH] OR Self-injurious behavior [MeSH] AND Nursing home [MeSH] OR Long-term care facility [MeSH]	PubMed	23,780	447	7
TITLE-ABS-KEY (Dementia) AND TITLE-ABS-KEY (Suicide or self-injurious behavior) AND TITLE-ABS-KEY (Nursing home or Long-term care facility)	Science Direct	13,965	125	2
“Dementia” AND “Suicide” OR “Self-injurious behavior” AND “Nursing home” OR “long-term care facility”	PubPsych	1865	52	1
TITLE-ABS-KEY (Dementia) AND TITLE-ABS-KEY (Suicide) OR (self-injurious behavior) AND TITLE-ABS-KEY (Nursing home) OR (Long-term care facility)	Cochrane library	18,299	64	2

We applied the Boolean method to combine the keywords and MeSH terms to synthesize a uniform search through the various databases. The last date of the search through all the databases was April 4, 2022. Additionally, keywords such as dementia, suicide, self-injurious behavior, nursing home, long-term care facility, and other synonyms such as neurocognitive disorder, self-destructive behavior, and assisted living were applied to the other databases. Furthermore, other publications in the reference list and related studies were also examined to see if they were relevant and could be included in this review.

Selection of Studies and Data Collection Process

Articles were screened and selected and 12 full-text articles meeting our inclusion criteria were reviewed independently by (NJ, RV, and OB) who discussed the design and characteristics of the studies to decide whether they could be included in the review. Then they extracted the relevant data which included: authors and publication year, study design, sample size and characteristic, location, years collected, and main findings.

Study Quality Assessment

Newcastle-Ottawa scale (NOS) was used to identify the eligible articles based on the kind of study for each publication. Two co-authors (NJ and NV) assessed the eligibility of the articles. Based on our quality score system, Newcastle-Ottawa, the 12 observational studies’ mean score was six.

The distribution of total scores for the Newcastle-Ottawa scale (NOS) for observational studies is indicated in Table 3.

**Table 3:** Summary of critical appraisal of included studies using the Newcastle-Ottawa quality assessment scale for observational studies * Selection: a) exposed truly representative of average, b) selection of non-exposed from the same community, c) exposure ascertained by secure record or interview d) demonstration of the outcome of interest not present at the start of the study; * Comparability: Comparability of cases and controls on the basis of the design or analysis controlled for confounder; * Outcome: a) follow up long enough for an outcome to occur b) complete follow up of all subjects c) subject lost to follow up unlikely to introduce bias; * Quality assessment: New castle Ottawa scale are converted to the AHRQ (Agency for Healthcare Research and Quality) standards. ---Thresholds for converting the Newcastle-Ottawa scales to AHRQ standards (good, fair, and poor): ***Good quality: 3 or 4 stars in selection domain AND 1 or 2 stars in comparability domain AND 2 or 3 stars in outcome/exposure domain ***Fair quality: 2 stars in selection domain AND 1 or 2 stars in comparability domain AND 2 or 3 stars in outcome/exposure domain ***Poor quality: 0 or 1 star in selection domain OR 0 stars in comparability domain OR 0 or 1 stars in outcome/exposure domain

Study ID [Ref]	Selection (Maximum 4)	Comparability (Maximum2)	Outcome (Maximum 3)	Quality assessment
Moon et al., 2021 [2]	***	*	***	Good
Seyfried et al., 2011 [3]	***		***	Good
McCarthy et al,.2013 [16]	***		***	Good
Temkin-Greener et al., 2020 [17]	***	*	***	Good
Günak et al., 2021 [18]	***	*	**	Good
Choi et al., 2021 [19]	**	**	**	Good
Nie et al., 2020 [20]	***	*	***	Good
Wongpakaran et al., 2012 [21]	***	*	**	Good
Gujral et al., 2021 [22]	***	**	**	Good
Holmstrand et al., 2021 [23]	***		**	Fair
Annor et al., 2019 [24]	***		**	Fair
Chappell et al., 2016 [25]	***		**	Fair

Result

The searches in PubMed, Pub Psych, Cochrane library and Science Direct databases revealed 57,909 articles. After screening based on exclusion criteria and achieving the complete text, the search in PubMed generated 447 articles, the searches in Pub Psych and Cochrane library generated 85 (53+32) additional articles, respectively, and the search in Science Direct provided another 156 articles. Of these, 137 duplicate articles were removed, and 539 selected articles were further excluded because they did not critically analyze the link between suicide and dementia in LTCs residents or relevant population. Ultimately, 12 articles were selected for inclusion in the present review. There were five cross- sectional studies, five cohort studies, two case-control studies, and one was systematic review.

**Figure 1:**
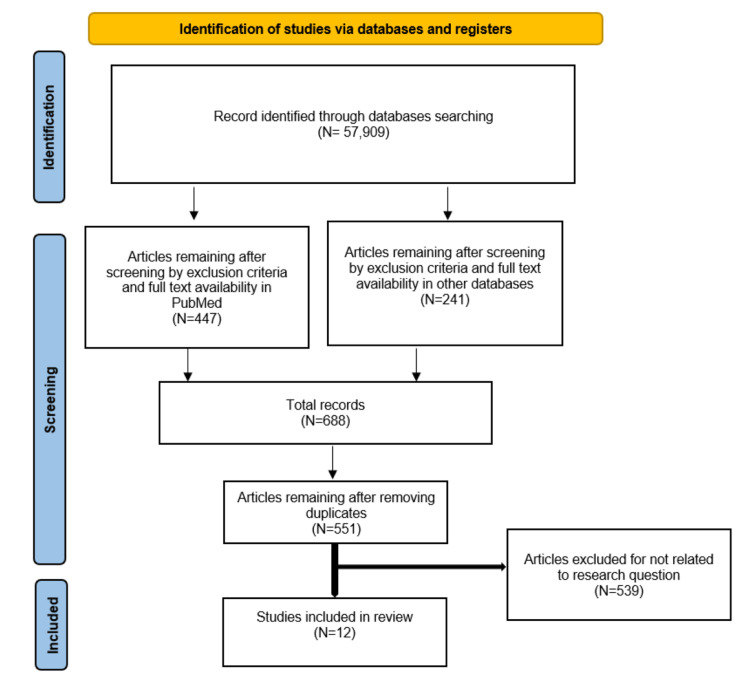
PRISMA 2020 flowchart for the search and selection process From: Page et al. The PRISMA 2020 statement: an updated guideline for reporting systematic reviews [15].

The main results of the search strategy used for selecting studies are shown in Figure 1.

In one study on 294,952 veteran affairs (VA) residents with dementia, an inpatient nursing home stay was associated with a lower risk of suicide vs. non-NH [3]. Another study has shown that suicide mortality in VA nursing home population was 107.2/100,000 which increased to 267.6/100,00 after six months of discharge. This study determined the hazard ratio of suicide among patients discharged from VA nursing homes with dementia at 0.66, lower than depression with 1.12 and severe mental illnesses with 1.02 [16].

Sungje Moon and colleagues 2021, mentioned that the suicide risk of 62,282 older adults with dementia and using long-term care in South Korea was about 0.256-times lower than those who did not use it, whereas it increased after the expansion of the dementia grading [2].

In 2020, Helena Temkin-Greener conducted a cross-sectional study on residents of 15,000 NHs in the USA. In this study, the probability of suicide ideation (SI) in residents with dementia at post-acute admission was 0.93 and decreased to 0.88 in long-stay admission group [17]. In contrast, Mia Maria Günak in 2021 demonstrated that the risk of suicide attempts among 147,595 residents of all VA medical centers in the USA was approximately 1.2 to 1.3 times higher in patients with mild cognitive impairment (MCI) or dementia diagnoses compared to the control group (patients without MCI or dementia) [18]. In addition, another study in 2020, declared that older patients with dementia in South Korea had an increased risk of suicide death compared to patients without dementia [19]. However, in two recent studies, the subjects were selected from the community, and the role of staying in LTCs on suicide risk has not been investigated.

The characteristics of included studies in this systematic review, are summarized in Table 4 and Table 5. 

**Table 4:** Characteristics of included studies in systematic review

Study Author, Year [Ref]	Study design	Sample size and composition	Sample characteristic	Time collected	Location	Main findings
Sungje Moon,2021 [2]	Cohort, retrospective	N= 62,282 from the “Older Adults Cohort DB” of the National Health Insurance Service	Older adults aged> 60 with dementia	2002-2015	South Korea	The suicide risk of older adults using LTCS (including facilities and in-home services) was about 0.256-times lower than those who did not use it. whereas it increased after the expansion of the dementia grading
Lisa S. Seyfried,2011 [3]	Cohort, retrospective	N=294,952 VA patients	patients ≥ 60 years old with the diagnosis of dementia, Male (97%)	2001-2005	USA	Suicide rate in study population: Inpatient NH stay: 2.5% versus outpatient psych visit 55.2% and outpatient non- psych visit 96.5% A history of inpatient psychiatric care was significantly associated with increased risk of suicide, while an inpatient nursing home stay which was associated with a lower risk of suicide
John F. McCarthy,2013 [16]	Cross-sectional	N=237,426 Persons Discharged Alive From 137 VA Nursing Homes	All ages, Male (96.9%)	2002-2008	USA	Suicide mortality in VA nursing home population ≥ 60 years old= 107.2/100,000 and after 6 months of discharge in same group = 267.6/100,00 which increased by age. Hazard ratio for suicide among patients discharged from VA nursing homes with dementia is 0.66, with depression is 1.12 and with serious mental illnesses is 1.02
Helena Temkin-Greener,2020 [17]	Cross- sectional	1,864,102 post-acute and 304,106 long-stay admissions to just over 15,000 NHs	Residents of NHs	7.2014- 6.2015	USA	predicted probability of SI in residents with dementia: post-acute admission=0.93 >post-acute discharge=0.92 > long stay admission=0.88 Residents with SI had a lower prevalence of moderate/severe cognitive impairment, but higher prevalence of depressive symptoms and moderate/severe aggressive behavior compared to residents without SI
Mia Maria Günak, 2021 [18]	Cohort study	N=147, 595 from all VA medical centers	Aged≥ 50 years old, 97.1 % were male and 86.1 % were non-Hispanic white	October 1, 2011-September 30, 2013, and follow-up through December 31, 2016	USA	The risk of suicide attempt was approximately 1.2 to 1.3 times higher in patients with MCI or dementia diagnoses compared to control group (patients without MCI or dementia)
Jae Woo Choi, 2021 [19]	Cohort, retrospective	N= 36541 from National Health Insurance Service–Senior Cohort data	older adults Aged ≥ 60 with newly diagnosed dementia (MMSE score ≤ 26	2004-2012	South Korea	Patients with dementia but without other mental disorders and patients with dementia and other mental disorders had an increased risk of suicide death compared to patients without dementia. Patients with dementia and schizophrenia, mood disorders or anxiety or somatoform disorders respectively, had an increased risk of suicide death compared to patients with those conditions but without dementia

**Table 5:** Characteristics of included studies in systematic review continued

Study Author, Year	Study design	Sample size and composition	Sample characteristic	Time collected	Location	Main findings
Yu Nie, 2020 [20]	Cross -sectional	N=817, living in nursing homes> 1 year	Older adults aged> 60 who don’t have severe physical or mental illness	Oct-Dec 2018	china	risk factors for suicidal ideation among elderly adults living in nursing homes were living in a rural area, infrequent visits from relatives, history of chronic disease, depression symptoms, reduced social support and ADL disability.
Nahathai Wongpakaran, 2012 [21]	Cross -sectional	N=81 from LTC facility residents	Aged 63-94	Feb-March 2011	Northern Thailand	40.7% were found to have cognitive impairment, and 23.5% met the criteria for current major depressive episodes. Though the majority was in the low-risk group, 32.1% were reported as being at risk of suicide.
Swathi Gujral,2021 [22]	Case -control	N=278 from community population	Aged ≥ 50 years old	2006-2018	USA	Both early-onset and late-onset suicide attempters performed worse on executive measures but late-onset attempters exhibited a broader range of low cognitive scores (global cognition, processing speed, memory) relative to non-suicidal depressed and non-psychiatric comparison groups
Cecilia Holmstrand,2021 [23]	Cohort study	N=1223 from community population	Older adults aged ≥ 65 years old, a primary diagnosis of dementia and a SMMSE score ≤24	2010-2013	8 European countries: Estonia, Finland, France, Germany, the Netherlands, Spain, Sweden and the United Kingdom	In the multivariate regression analysis, country of origin, moderate stage of the dementia, depressive and delusional symptoms, and anti-dementia medication were significantly associated with suicidal ideation. Over time, suicidal ideation decreased from severe to mild or became absent in 54% of the persons with dementia.
Francis B. Annor, 2019 [24]	Cross- sectional	N=91 from Georgia Vital Records and Georgia Violent Death Reporting System	Patients with dementia who died by suicide	2013-2016	Georgia, USA	Suicide rate among persons with dementia was 9.3 per 100 000 person-years overall and substantially higher among those diagnosed in the past 12 months (424.5/100 000 person-years)
Phillip Chappell,2016 [25]	Online survey systematic review	N=204 respondents who completed online survey	Psychologist, psychiatrist, neurologist and nurses who personally conducted SI/ SB assessments during the course of clinical trials	March 2013	North America and Europe (83.4%) and the remainder from Asia, Latin America, and Mideast/Africa	suicidal ideation and behavior do occur in clinical trials of patients with MCI or dementia. However, the reported occurrence of Suicide Behavior (SB) and Completed Suicide (CS) appears to be lower than Suicide Ideation (SI) and may decline further with increasing severity of dementia.

Discussion

The primary finding from this review is that in last 12 years, few studies have been conducted to determine the suicide risk among residents of LTCs who have dementia. Three studies have been led on VA patients who were predominantly male (~ 97%), so the results cannot be generalized to all genders [3,16,18]. 

Six studies examined the risk of suicide in nursing home residents [2,3,16,17,20,21]. Among these studies, four articles carefully examined the relationship between dementia and suicide, and all confirmed the hypothesis that staying in LTCs reduces the risk of suicide in patients with dementia [2,3,16,17]. The reason could be facilitated contact with peers, more excellent monitoring of daily activities, more contact with health and mental health professionals, and presumably less access to lethal means of suicide in LTCs [26]. Other important factors include life skills (problem solving skills and coping skills, ability to adapt to change), self-esteem and a sense of purpose or meaning in life and cultural, religious, or personal beliefs that discourage suicide [27].

Assessing the risk of suicide at long-term-care facilities can be dilemma. Since nursing home residents might be at a higher risk of suicide than those living independently in the community because of having higher rates of chronic illnesses, functional impairments and psychiatric conditions, it’s often argued that nursing home residents should be at a lower risk because of early suicide risk assessment by professionals, 24 hours a day staff presence and less access to dangerous items such as guns [28].

In a study, it was noted that the occurrence of completed suicide (CS) and suicide behavior (SB) is lower than suicide ideation (SI) in demented patients [25]. This report reveals the need for screening in these patients in order to find people with SI, which is the foreground of the occurrence of CS. Furthermore, we found that most studies in this field, have investigated the risk factors of suicide and only a few of those have examined the protective factors of staying in LTCs as well as the role of psychotherapy, problem solving skills and coping skills on reducing the suicide risk in dementia. Hence, further studies in this filed are strongly recommended.

Methods of suicide in LTCs

In one study, the most common methods of suicide in LTC settings included hanging (five studies), jumping (three studies), drug overdose (two studies), firearm (two studies), wrist slashing, asphyxiation, refusing to eat or drink, medication refusal, drowning, and self-poisoning [26].

Another study mentioned that the most common methods of suicide among nursing homes residents were firearm (22%), hanging/suffocating (22%), fall (17%), cutting (15.3%), poisoning (11.9%) respectively [29]. However, when compared to methods of suicide in the community, cases in LTC facilities were less likely to involve firearms and 2.6 times more likely to involve falls [30].

The standard methods of suicide in long term care facility residents in Sweden included poisoning, hanging/strangulation and suffocation, jumping from a high place, drowning and submersion, smoke/fire and flame, sharp objects and shotgun, respectively [31]. While the most common methods of suicide among nursing home residents in Australia were hanging (31.9%), fall from height (17.0%), and plastic bag asphyxia (14.2%) [32].

Methods of suicide in dementia

In one study that compared the methods of suicide in patients with dementia with a control group, self-poisoning, drowning, hanging, jumping (height/moving vehicle), suffocating and cutting were the most common methods, respectively [33].

 A study conducted by Lisa Seyfried and colleagues in 2011 demonstrated that the methods used in the vast majority of suicides in demented patients were firearm (72.6%), self-poisoning and hanging (9.5% each), harm with a sharp object (2.9%), jumping from a high place or moving object (2.4%), drowning (1.2%) or self-immolation (0.4%) [3].

Another study determined the firearm (55%), unknown (31.8%) and poisoning (13.2%) as the mechanism of suicide among patients with dementia in Georgia [24].

Purandare et al., in the study of a nine-year national clinical survey in England and Wales, observed that self-poisoning was the most common method of suicide in the dementia group, accounting for almost a third of deaths, followed by drowning (19%) and hanging (17%) [34].

At a global level, older adults tend to choose the most lethal methods, especially hanging [35]. However, in the USA, firearm was the most common method used in 2019 in all age groups, accounting for about half (n =23,941) of all deaths due to suicide [36].

Risk and protective factors of suicide in dementia residing in LTCs

While some suicide risk factors such as male gender, non-Hispanic white race, depression, previous history of suicidal behavior, multiple medical conditions, pain and sleep disturbances and low social and family support in people with dementia are same as other people, some have shown to have contradictory effects on people living in nursing homes compared to people in the community. For instance, married people in the community have a lower risk of suicide because of emotional support from their spouses. In contrast, a study has demonstrated that residents in nursing homes away from their partners have a higher suicide risk [37].

Few studies endorsed that the group of patients with more severe cognitive and/or physical limitations who would thus have more difficulty preparing and executing a suicide plan have a lower risk of suicide [2,3,23]. Similarly, a diagnosis of schizophrenia, an illness also associated with executive and planning deficits, was potentially protective [3]. However, another study stated that patients with dementia and schizophrenia, mood disorders, anxiety or somatoform disorders had an increased risk of suicide death [19].

The risk and protective factors of suicide in nursing home's residents with dementia are summarized in Table 6. 

**Table 6:** The protective and risk factors of suicide among demented residents of LTCs

Risk factors [Ref]	Protective factors [Ref]
New dementia diagnoses within 1 year [3,19,24]	nursing home admissions/use of LTCs [2,3,17]
Comorbid depression [3,22,24]	comorbid schizophrenia [3]
Mild dementia/early stage [2,23]	Late stage dementia/severe cognitive impairment [2,3,23]
Non-Hispanic White [3,18]	Physical limitations/fully dependent [2,3]
Younger age [2,3,19,24]	Older age [2,18,19,24]
With comorbidities [19]	Without comorbidities [19]
Low social support [20]	Positive social relationships and support [20,38]
Low frequency of visits from relatives [20]	High frequency of visits from relatives [20]
Living in urban area [20]	Living in Rural area [20]
previous history of suicidal behavior [18]	less access to lethal means of suicide [26]
Male [2,3,24]	More monitoring by mental health professionals [26]
Anxiety and aggressive behavior [3]	
Presence of pain/sleep disturbances [17,18]	
History of inpatient psychiatric hospitalizations [3]	
prescriptions for antidepressants and anxiolytics [3]	
Transition to the LTC facility [39]	

Limitation

Limitations of this study include lack of access to the full text of related articles despite attempting to reach out to the author and/or correspondent author. At the result, we lost a number of publications that could have increased the strength of our study and helped us draw better conclusions. Other limitation is existing limited number of studies conducted in recent years related to suicide in demented patients living in long-term care facilities. We also reviewed articles from the last 12 years written in English, and so the further of other useful related articles were removed from our assessment.

## Conclusions

Existing research on suicide risk in LTCs residents with dementia is limited. Due to the increase in dementia rates that require people to reside in NHs and on the other hand, considering the multiple risk factors of suicide in the elderly people residing in such places, paying greater attention to the screening system to find people with SI, which is the foreground of the occurrence of CS as well as training related professional in psychotherapy, problem solving skills and coping skills to reduce the suicide risk in dementia for identifying people at risk and performing preventive therapeutic and behavioral interventions is well felt.
